# Combined bio-logging and stable isotopes reveal individual specialisations in a benthic coastal seabird, the Kerguelen shag

**DOI:** 10.1371/journal.pone.0172278

**Published:** 2017-03-06

**Authors:** Elodie C. M. Camprasse, Yves Cherel, John P. Y. Arnould, Andrew J. Hoskins, Charles-André Bost

**Affiliations:** 1 School of Life and Environmental Sciences, Deakin University (Burwood Campus), Geelong, Victoria, Australia; 2 Centre d’Etudes Biologique de Chizé (CEBC), Centre National de la Recherche Scientifique, UMR 7372 du CNRS-Université de La Rochelle, Villiers-en-Bois, Deux-Sèvres, France; 3 CSIRO Land and Water, Canberra, Australian Capital Territory, Australia; Friedrich-Schiller-Universitat Jena, GERMANY

## Abstract

Individual specialisations, which involve the repetition of specific behaviours or dietary choices over time, have been suggested to benefit animals by avoiding competition with conspecifics and increasing individual foraging efficiency. Among seabirds, resident and benthic species are thought to be good models to study inter-individual variation as they repetitively exploit the same environment. We investigated foraging behaviour, isotopic niche and diet in the Kerguelen shag *Phalacrocorax verrucosus* during both the incubation and chick-rearing periods for the same individuals to determine the effect of sex, breeding stage, body mass and morphometrics on mean foraging metrics and their consistency. There were large differences between individuals in foraging behaviour and consistency, with strong individual specialisations in dive depths and heading from the colony. Stable isotopes revealed specialisations in feeding strategies, across multiple temporal scales. Specifically, individuals showed medium term specialisations in feeding strategies during the breeding season, as well as long-term consistency. A clustering analysis revealed 4 different foraging strategies displaying significantly different δ^15^N values and body masses. There were no sex or stage biases to clusters and individuals in different clusters did not differ in their morphology. Importantly, the results suggest that the different strategies emphasized were related to individual prey preferences rather than intrinsic characteristics.

## Introduction

According to the optimal foraging theory, an individual should select a specific foraging strategy that maximizes its net energy intake per unit of time while minimizing other costs such as predation risk [1]. Foragers would be expected to maximize foraging efficiency even more when provisioning young as foraging effort is increased to meet the energy requirements of the offspring [2]. Individual specialisations have been suggested as a way to avoid competition with conspecifics and to increase individual foraging efficiency (including prey finding, handling, and digesting) [3]. They have been linked with greater body condition, fitness or reproductive output in some species [4, 5].

Individual specialisations in foraging involve the repetition of specific behaviours or dietary choices over time, and have been, until recently, poorly investigated [3, 6, 7]. Individual specialists can be defined as “individuals whose niche is substantially narrower than their population’s niche for reasons not attributable to their sex, age or discrete morphological group” [6]. It is of importance to identify the mechanisms generating inter-individual variation and study the wider implications of variation in foraging behaviour if we are to understand trophic relationships between the animals and their environment [6, 8–10]. In addition, these variations in foraging behaviour may have substantial impacts on ecological processes and foraging dynamics [9].

Individual specialisations are reported across a wide range of taxonomic groups including molluscs, crustaceans, insects, fishes, reptiles, amphibians, birds and mammals [6]. Consistency in animal behaviour and niche has also been reported in a wide range of contexts: mate choice [11, 12]; nesting behaviour [13]; wintering strategies [14]; feeding strategies [15]; space use [3]; trophic levels [16, 17]; responses to environmental variables [18]; and boldness [19].

Determining the temporal consistency of individual specialisations requires longitudinal studies involving repeated observations on individuals over time [6]. Seabirds generally nest in colonies and are central-place foragers during the breeding season and, therefore, offer the possibility for such longitudinal studies. Indeed, many seabirds can be accessed repetitively throughout the breeding season and also across years as they display a high level of nest fidelity. In addition, collecting data from multiple members of the same colony allows the level of variation in diet and behaviour between individuals that arises from specialisation to be determined as animals have access to the same resources and are exposed to the same environmental conditions [20].

Cormorants are inshore feeders, foot-propelled pursuit-divers, feeding on a wide range of inshore benthic or pelagic prey in a limited ecological niche [21–23]. Their body plan implies high flight and diving costs [24, 25]. In addition, because they are air-breathers and have a wettable plumage, the amount of time they can spend diving is limited [26]. The Kerguelen shag, *Phalacrocorax verrucosus* (Cabanis 1875), is a member of the 13 species so-called blue-eyed shag complex [27, 28], which represents one of the main top predators to feed on the fish community in the coastal areas of the Antarctic and subantarctic territories [29].

Flexibility in feeding habits has been reported for various species of cormorants, and it has been argued that this flexibility plays an important role in maximizing their food intake [22]. For example, species in the ‘blue-eyed shag complex’ can exhibit a high intra- and inter- individual variation in diving behaviour and prey choice, as well as inter- individual and inter-sexual differences [7, 30, 31]. Species in this group can display strong individual specialisations that can be maintained across years [7, 32–34]. These philopatric, mostly benthic foraging birds are well suited to answer such questions as they are long-lived species, repetitively breed and forage in the same locations. Kerguelen shags show dietary specialisations [23, 32], but the consistency in their space use and diving behaviour has not been investigated in detail.

The aims of the present study were to (1) quantify and identify the factors influencing consistency in diving behaviour, space use, and diet in Kerguelen shags at different temporal scales, as well as to (2) study the links between foraging behaviour, consistency in foraging, diet, and morphometry. Different complementary approaches, representing different timescales, have been used to do so as no single timescale may provide a complete and accurate picture of the level of individual specialisation [35]. Snapshot methods such as regurgitate analysis provide essential diet information, while stable isotope analysis on tissues with different turnovers allow to examine the diets and foraging habitats across longer timescales, and GPS/TDR provide a fine-scale representation of foraging behaviour and allow the identification to foraging sites [10].

## Materials and methods

### Instrumentation

Fieldwork was conducted at the Pointe Suzanne Kerguelen shag colony (49°26’S, 70°26’E), Kerguelen Island, southern Indian Ocean, during the 2014/15 breeding season. This study was approved by the ethics committee of the French Polar Institute (Program IPEV 394, resp. C.A. Bost) and therefore meets ethics guidelines. All animals in this study were cared for in accordance with its guidelines. Sampling occurred during two sessions. During the first session, a total of 20 individuals (both partners from 10 nests) were instrumented with GPS data loggers (I-gotU GT120, Mobile Action, Taiwan; 44.5 x 28.5 x 13 mm, 22 g in air corresponding to *ca* 1% of mean body mass) for 3–6 d at the end of incubation/early chick rearing (26-Nov-2014 to 10-Dec-2014; hereafter incubation), when chicks were no older than a week. During the second session, a total of 22 birds (both partners from 11 nests) were instrumented for 3–12 d during late chick rearing (6-Jan-2015 to 18-Jan-2015; hereafter chick-rearing), of which 10 birds had previously been sampled and were deployed only with GPS data loggers while the remaining 12 individuals were equipped both with GPS data loggers and time-depth recorders (TDR, LAT1800S, Lotek Wireless Inc.; 36 x 11 x 7.2 mm, 4.8 g in air corresponding to *ca* 0.2% of mean body mass). All chick-rearing instrumented birds had a single chick, except for one nest, which had two chicks at the beginning of deployments but lost one of them within a few days. GPS loggers were programmed to sample positions every one min at incubation and every 2 min in chick-rearing. The TDR units were set to record depth and temperature at 1 s intervals.

Individuals were captured at the colony using a noose attached to a fishing pole, weighed in a cloth bag using a suspension scale (± 25 g, Pesola AG Baar, Switzerland), and banded with an individually numbered coloured plastic ring on one leg and an individually numbered metal ring on the other for identification. The GPS loggers, removed from their housings and encased in heat shrink plastic for waterproofing, and the dive recorders were attached to the back feathers using waterproof tape (Tesa 4651, Quickborn str 24, Hamburg 20253, Germany) and cyanoacrylate glue (Loctite 401, Prism, Instant Adhesive, Hempstead, Hertfordshire, HP2 4RQ UK).

Individuals were recaptured 3–18 days later using the methods previously described. The data loggers were removed and individuals were weighed again and morphometric measurements (bill length, bill width, head length, wing length and tarsus length) were taken with a vernier caliper and metal ruler (± 0.05 and 1 mm, respectively). In addition, 3–6 dorsal dark contour feathers from between the wings were plucked and a blood sample (0.5–1.5 mL) was obtained by venipuncture of a tarsal vein. Spontaneous regurgitations during handling were collected for later analysis of prey remains. Handling times ranged 15–20 min during which the bird’s head was covered with a hood to reduce stress.

Due to battery malfunction or flooding of loggers, GPS positions were obtained from only 36 deployments (29 different birds in total), each of which comprised 3–18 trips. Out of these deployments, 15 were carried out during incubation, and 21 during the chick-rearing period. Seven birds were used during both breeding stages, at an approximately one-month interval.

### Isotopic and dietary analyses

The isotopic method was validated in the southern Indian Ocean, with δ^13^C values of seabirds indicating their foraging habitats [36, 37], and their δ^15^N values increasing with trophic level [38]. Isotopic values (details in [39]) were measured on whole blood (hereafter blood) and contour feathers of shags. The rationale is that the two complementary tissues integrate different periods of information, due to different turnover times. Blood is a metabolic active tissue that integrates a period of weeks before sampling, whereas feathers reflect the diet at the time they were grown, because keratin is inert after synthesis. Here, blood and feathers collected during the breeding period reflect the breeding period itself and the previous post-breeding moulting period that took place almost one year before the study, respectively. In the laboratory, blood samples were freeze-dried and powdered. Lipid extraction was not necessary as the C:N mass ratio was < 3.5 for all blood samples [40]. A single contour feather per bird was cleaned of surface lipids and contaminants using a 2:1 chloroform: methanol bath, air-dried and cut into small pieces. Nitrogen and carbon isotopic ratios were measured with a continuous-flow isotope-ratio mass spectrometer (Thermo Scientific Delta V Advantage) coupled to an elemental analyser (Thermo Scientific Flash EA 1112). Results are presented in the usual δ notation relative to Vienna PeeDee Belemnite (PDB) for carbon and atmospheric N_2_ (AIR) for nitrogen. Replicate measurements of internal laboratory standards (acetanilide) indicated measurement errors < 0.15 ‰ for both δ^13^C and δ^15^N. Blood isotopic values were obtained from 32 individuals and feather values from 31 individuals (including 9 shags that were sampled twice, at incubation and then in chick-rearing; 12 of those individuals had GPS and TDR data over consecutive trips and 15 of those had GPS data only over consecutive trips).

Regurgitate samples were stored frozen until processing in the laboratory. They were first defrosted and the fresh and accumulated fractions were weighed separately. The fresh fraction was sorted into different prey categories (annelids, cephalopods and fish) that were weighed separately. Items were identified to species level when possible, according to [41] for fish, and to a reference collection, i.e. bones and otoliths for fish, chitinized beaks for cephalopods and jaws for annelids. Standard length (SL) was measured in the few intact fishes. Otherwise, otoliths were measured (precision ± 0.01 mm) and SL estimated from allometric equations [42, 43].

### Data processing and statistical analyses

All data analyses were conducted in the R Statistical Environment version 3.2.0 [44]. GPS records for each individual were visually inspected and individual trips were determined. On average, GPS points during foraging trips were obtained every 3.1 minutes for GPS loggers set to record every minute, and every 3.8 minutes for the ones set to record every 2 minutes. The *diveMove* package was used to apply a speed filter to the GPS data to remove erroneous locations with a threshold of 18 m·s^-1^, and obtain summaries of diving metrics from TDR records (only dives deeper > 1 m were considered). Means of diving behaviour metrics and coefficients of variation (CV) per individual were calculated for dive duration, depth, and sum of vertical distance.

GPS records were linearly interpolated to the 1 s intervals in the *adehabitatLT* package [45] to provide spatial information for the dive records. Furthermore, the packages *trip* [46] and *sp* [47] were used to obtain summaries of at-sea movements and to calculate the number of grid cells used (1x1 km grid cells) per trip. Means and CVs per individual and per stage were calculated. Heading for each trip was calculated as the angle between the colony and the most distal point of the tracks, and standard deviation in heading was calculated for each individual using the *circular* package.

An index of consistency in space use was calculated for each animal within each stage. For each trip the number of grid cells used by the individuals were identified. The number of shared grid cells for each pair of trips (e.g. trip 1 and trip 2, trip 2 and trip 3, trip 1 and trip 3 etc.) was determined and the average of these calculated. This number was then divided by the average number of grid cells used per trip. To assess whether spatial information alone could reflect consistency in foraging locations, this index was compared for the GPS derived tracks and the dive locations alone. There was a significant positive correlation between the consistency index calculated from GPS data alone and diving locations obtained using GPS and TDR (R^2^ = 0.74, P < 0.001). Therefore, it was considered that consistency in spatial space use was representative of consistency in foraging space use. Different grid cell sizes were used to calculate the index of consistency in space use (from 0.5x0.5 km to 5x5 km) to check the influence of grid cell size on our estimate of spatial consistency. Indices obtained, regardless of cell grid sizes, were highly correlated.

In order to investigate the factors influencing dive behaviour, spatial use and consistency in foraging behaviour, linear mixed effects models were fitted, using the *nlme* package [48]. For all models, backward-stepwise model selection was used to select the most parsimonious model [20]. First, the most appropriate random effects structure was identified with the restricted maximum likelihood (REML); then the best fixed effects structure was determined using maximum likelihood (ML) before refitted the selected model with REML to estimate the model parameters.

As maximum depths, total vertical distances and dive durations were correlated, we used maximum depths as a representative explanatory variable to investigate the drivers of diving behaviour. Specifically, maximum depth was used as a response variable, sex or mass as explanatory variables, trip nested within individuals nested within pairs as random effects, and a power variance structure. To investigate the effect of sex on dive behaviour consistency, we selected the coefficient of variation in maximum depth as explanatory variable, as this metric was correlated with the coefficients of variation for total vertical distances and for dive durations. The model included sex as an explanatory variable, and individual as a random effect. In order to quantify how specialised shags were in diving behaviour, we used a variance components analysis to calculate the variance, standard deviation and proportion of total variance occurring at the levels of individual, and trip within individual using the R package ape [49] following [20]. An estimate of individual specialisation is given by the proportion of variance explained by the individual variance component [6, 20, 50].

To understand the influence of sex and stage on spatial metrics, total distance travelled was used as an explanatory variable as it was correlated with maximum distance and trip duration; sex, stage and their interaction were used as explanatory variables, individual was used as a random effect and a sex and stage identity variance structure was applied. Heading to most distal point was included as the response variable in a second similar model, except with individual nested within pair as a random effect. To understand the effects of morphology on these two metrics, the least correlated morphometrics (i.e head length, wing length and mass at deployment) were used as explanatory variables in models containing individual as a random effects and a power variance structure.

To look at whether sex and stage influenced the consistency in spatial use, we used the coefficient of variation in total distance as a response variable. Indeed, this metric was correlated with the coefficients of variation in maximum distance and trip duration, with the standard deviation in heading and with the index of spatial use consistency. The model included sex, stage and their interaction as fixed effects, individual as a random effect, and a sex and stage identity variance structure. In order to quantify how specialised shags were in spatial use, we used a variance components analysis as described above. This was not done separately for each sex, however, because no difference in spatial use consistency was detected between sexes.

In order to identify differences in foraging strategies, an agglomerative hierarchical clustering analysis with Euclidian distance and Ward’s linkage criterion [51] was performed on mean and CVs of trip spatial metrics for each individual within stages. The function “HCPC” of the *FactoMineR* package was used to determine the appropriate number of clusters [52]. Data on diving behaviour were not included in this clustering analysis as only a third of individuals were instrumented with a TDR and only in a single breeding stage. Linear mixed effects models were used to determine if cluster affiliation had a significant impact on mass and isotopic values. Null models (including no fixed factors and individual nested within breeding stages as random factors) were compared with models additionally including cluster number as a fixed factor. Significant differences between both models fitted by maximum likelihood indicated that clusters varied significantly in terms of the response variable of interest. *Post hoc* Tukey HSD multiple comparison tests were then conducted to determine more specifically which clusters differed in the *multcomp* package [53].

To look at the effect of sex and stage on stable isotopes, linear mixed models were used with blood δ^13^C or blood δ^15^N values as response variables. In the first case and second case, the random effects were individual and individual nested within pair, respectively. In order to assess dietary specialisations, linear mixed effects models were used, with either the isotope values during chick-rearing as response variables and the isotope values during incubation as explanatory variables, or feather isotope values as response variables and blood values (averaged between samples when repeat samples were obtained) as explanatory variables. Pair was included as a random effects in those 4 models. Unless stated otherwise, values presented are means ± SE.

## Results

### Foraging behaviour and its consistency

**Fig 1:**
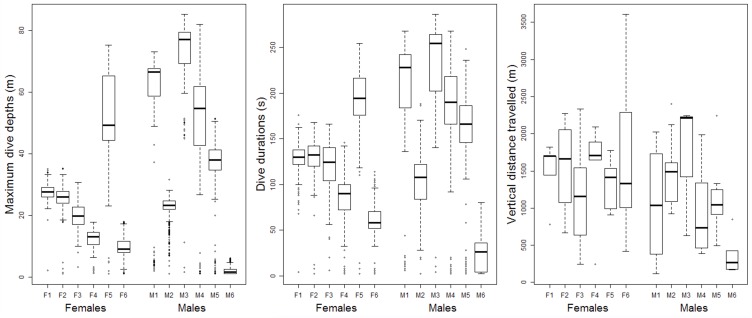
Boxplots of maximum dive depths, dive durations for all dives, and vertical distance travelled for all trips of individual male and female Kerguelen shags instrumented from the Pointe Suzanne colony, Kerguelen Islands (n = 6 males and 6 females).

**Fig 2:**
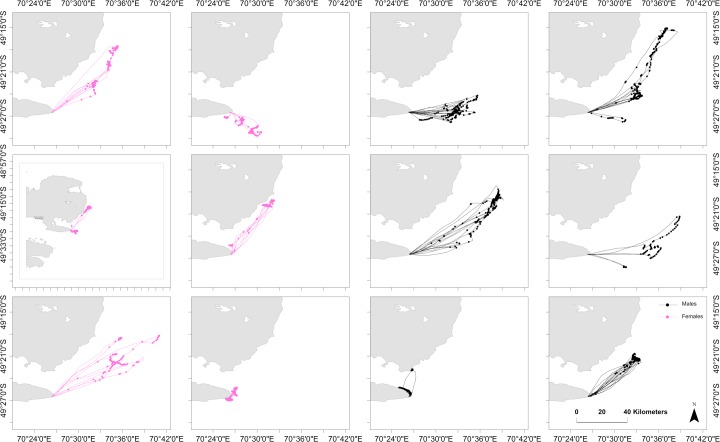
Individual tracks and dive locations for all trips of individual Kerguelen shags instrumented during the chick-rearing period at the Pointe Suzanne colony, Kerguelen Islands (n = 12).

Four to ten trips per individual were obtained (n = 76 trips) from birds equipped with both GPS and TDR during the chick-rearing period. A total of 5679 dives were recorded with individuals displaying large variations in the means and CV of diving behaviour (S1 Table). There was a trend for males to dive deeper than females and be less consistent in their maximum depths (Fig 1). However, a sex effect on maximum depth could not be detected (LME: P = 0.10, df = 1, F = 3.93). Dive depths increased with mass at a rate of 41.0 ± 11.24 m·kg^-1^ (LME: P = 0.01, df = 1, F = 13.31). Sex influenced the CV in maximum dive depths, with CV being higher by 0.13 ± 0.05 in males compared to females (LME: P = 0.02, df = 1, F = 7.90). There was substantial inter-individual variation in space use but, in general, all animals tended to dive at the section of the foraging trip most distant from the colony (Fig 2).

**Fig 3:**
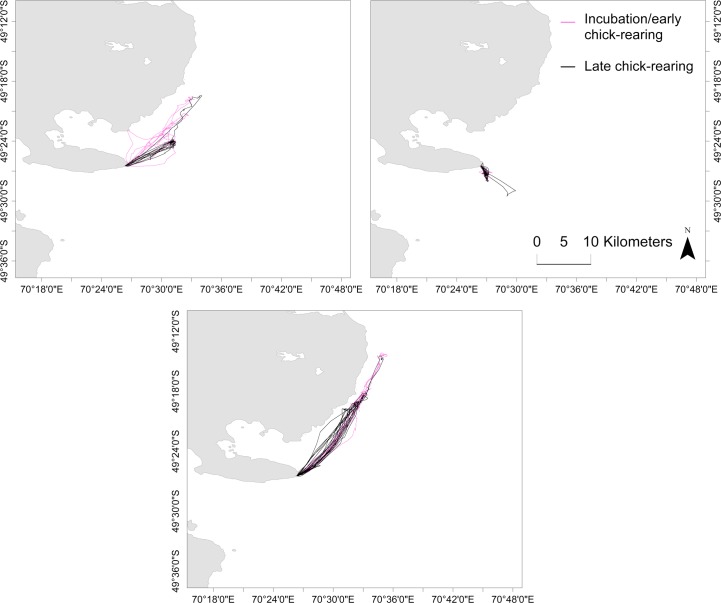
Tracks for Kerguelen shags instrumented at the Pointe Suzanne colony, Kerguelen Islands during the incubation and chick-rearing periods (subset of 3 representative birds).

There were strong differences between individuals in mean trip metrics (Fig 2, S2 Table). Kerguelen shags were sexually dimorphic (S3 Table). Overall, sex and breeding stage did not influence individual foraging behaviour and consistency in a predictable manner (Table 1). The best model to explain total distances travelled, however, only included stage as an explanatory variable, with total distances travelled 6.32 ± 2.21 km higher at incubation, compared to chick-rearing (LME: P = 0.005, df = 1, F = 8.22). In contrast, heading to most distal point was not explained by sex or breeding stage as the best model to explain heading did not include either sex or stage. In terms of morphology and mass, mass best explained total distance, which increased with bird mass at a rate of 16.45 ± 7.47 m·kg^-1^ (LME: P = 0.03, df = 1, F = 4.85), while heading to most distal point was not influenced by mass, wing length or head length. Sex and stage did not influence the CV for total distances travelled. In addition, birds instrumented during both incubation and chick-rearing used the same foraging areas (Fig 3), suggesting individuals displayed consistent behaviours over the whole breeding season.

**Table 1:** Summary of trip metrics for Kerguelen shags instrumented at the Pointe Suzanne colony, Kerguelen Islands, separated by sex and breeding stage (values are means ± SD).

Variable	Females		Males	
	Incubation (n = 8, 35 trips)	Chick-rearing (n = 10, 61 trips)	Incubation (n = 7, 48 trips)	Chick-rearing (n = 11, 90 trips)
Trip duration (h)	5.9 ± 1.8	6.1 ± 3.1	5.4 ± 2.9	5.1 ± 2.6
Maximum distance (km)	10.2 ± 6.5	9.0 ± 6.7	9.5 ± 8.0	11.1 ± 5.5
Total distance (km)	26.6 ± 14.0	22.7 ± 15.2	25.0 ± 19.5	29.6 ± 20.3
Heading (°)	47.6 ± 0.15	60.8 ± 0.36	75.4 ± 0.44	55.9 ± 0.26
Index of space use consistency	0.6 ± 0.13	0.5 ± 0.18	0.4 ± 0.20	0.5 ± 0.16

### Foraging strategies, diet and specialisation

The shags instrumented in this study displayed strong degrees of individual specialisations (Table 2). Males were less specialized than females in diving behaviour (maximum depths: proportion of variance explained by individual component = 41.7 and 85.1% respectively). Specialisations in spatial use were also high (proportion of variance explained by individual component = 84.5 and 72.7%, for total distances travelled and headings to most distal point, respectively) (Table 2).

**Table 2:** Variance component analysis of Kerguelen shag dive depths, total distances travelled and headings to most distal point.

Variance component	Males			Females		
	σ^2^	Σ	σ^2^%	σ^2^	Σ	σ^2^%
Maximum depths (n = 6 males, n = 6 females)						
Individual	127.6	11.3	41.7	163.0	12.8	85.1
Trip	171.6	13.1	56.1	27.5	5.2	14.4
Residual variation	6.58	2.6	0.02	1.1	1.0	0.01
Males (n = 14), females (n = 15)						
	σ^2^		Σ		σ^2^%	
Total distance travelled						
Individual	137.9		11.7		84.5	
Stage	24.9		5.0		15.3	
Dive	0.4		0.6		0.002	
Heading to most distal point						
Individual	1482.1		38.5		72.7	
Stage	0.0		0.01		0.0	
Trip	556.1		23.6		0.27	

**Fig 4:**
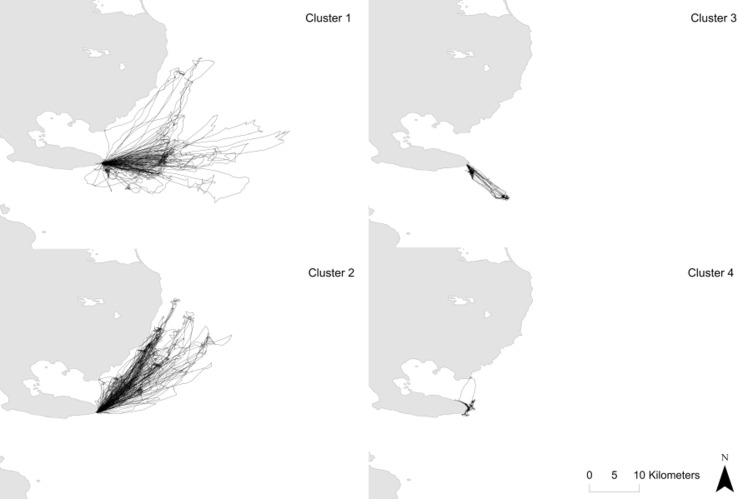
Successive tracks of all individuals in each cluster (for definition, see text) for Kerguelen shags instrumented at the Pointe Suzanne colony, Kerguelen Islands. Cluster 1 (n = 15 individuals), cluster 2 (n = 10 individuals), cluster 3 (n = 2 individuals), cluster 4 (n = 2 individuals).

Results of the clustering analysis incorporating the means of space use variables and their CVs grouped individuals into 4 clusters corresponding to different foraging strategies (Fig 4). Each strategy differed in mean heading, consistency in space use, standard deviation in heading and total distance travelled (Table 3). Individuals from clusters 1 and 2 (n = 15 and 10, respectively) exploited foraging areas to the east and to the north, north-east of the colony, respectively. These 2 clusters had the highest total distances travelled and indices of space use consistency. Individuals from cluster 3 (n = 2) showed intermediate travelled distances and foraged to the south, south-east of the colony. Individuals from cluster 4 (n = 2) foraged very close to the colony, to the north or south. Both clusters 3 and 4 had individuals that were much more consistent in their space use compared to the remaining clusters. Due to small sample size in each cluster, we could not test whether clusters differed in diving behaviour.

**Table 3:** Differences in space use metrics, mass and blood δ^15^N values between clusters identified for Kerguelen shags instrumented at the Pointe Suzanne colony, Kerguelen Islands (means ± SE).

Variable	Cluster 1	Cluster 2	Cluster 3	Cluster 4
Total distance (km)	45.4 ± 5.4	33.5 ± 4.7	16.4 ± 7.6	2.1 ± 0.5
Mean heading (°)	35.6 ± 2.4	82.2 ± 4.0	155.0 ± 5.7	-21.2 ± 9.5
Index of space use consistency	0.61 ± 0.0	0.36 ± 0.1	0.72 ± 0.1	0.57 ± 0.0
Standard deviation in heading	0.17 ± 0.0	0.46 ± 0.1	0.08 ± 0.0	0.78 ± 0.6
Mean mass (kg)	2.2 ± 0.0	2.4 ± 0.1	2.1 ± 0.1	2.0 ± 0.1
Blood δ^15^N (‰)	15.0 ± 0.2	14.1 ± 0.3	13.9 ± 0.6	16.1 ± 0.8

Individuals instrumented in both breeding stages were always classified in the same cluster confirming the existence of stereotyped behaviours at one-month interval. There were no sex or breeding stage biases to clusters. Lastly, members of different clusters did not vary significantly in morphology. Importantly, there were differences in body mass between clusters, with individuals from cluster 2 being significantly heavier (but not structurally larger) than individuals from all other clusters (LME: df = 3, F = 3.97, P = 0.02). A small sample size and unequal representation of individuals in each cluster, prevented us from testing whether clusters differed in foraging success (mass gains of adults during deployment at incubation or mass gains of chicks during chick-rearing).

**Fig 5:**
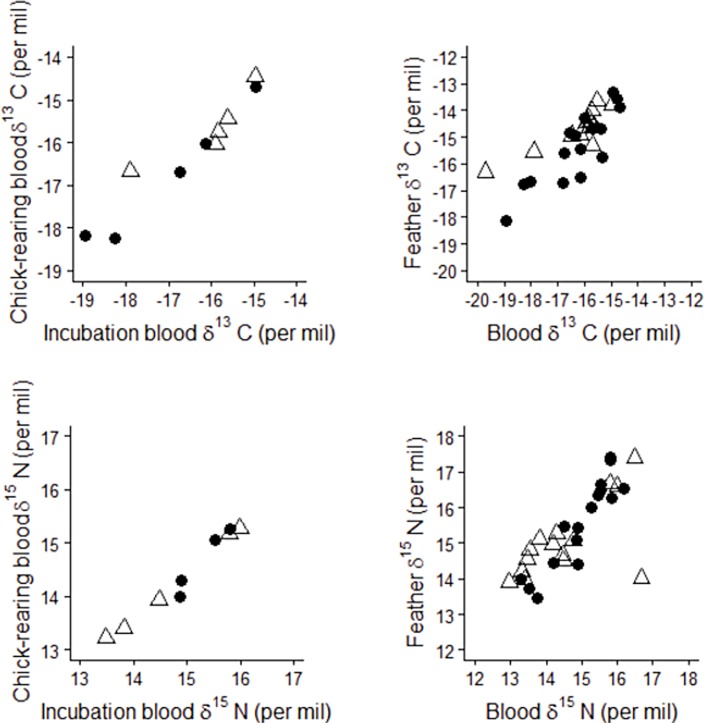
Medium- and long-term specialisations in Kerguelen shags sampled at the Pointe Suzanne colony, Kerguelen Islands, as shown by the correlations between incubation and chick-rearing blood δ^13^C and δ^15^N values, and between blood and feather δ^13^C and δ^15^N values, respectively (n = 10 and 30, respectively). (●) males, (Δ) females.

Blood δ^13^C and δ^15^N values averaged -16.3 ± 1.3 and 14.6 ± 1.0 ‰, respectively (C:N mass ratio: 3.39 ± 0.04, n = 41). Large ranges in blood isotopic values reflected foraging variation between individuals during breeding (S4 Table), with differences between the lowest and highest values amounting to 5.5 and 3.8 ‰ in δ^13^C and δ^15^N, respectively. Blood δ^13^C and δ^15^N values during incubation and chick-rearing (sampling at 32–44 days interval) were highly significantly positively correlated (90 ± 9% for δ^13^C, df = 1, F = 102.75 and 93 ± 6% for δ^15^N, df = 1, F = 246.94, respectively; Fig 5). Blood δ^13^C values were influenced neither by sex nor breeding stage. Stage was the only variable to significantly influence blood δ^15^N values, which were 0.56 ± 0.07 ‰ higher during incubation (LME: breeding stage, P<0.001, df = 1, F = 59.93). Mass was the only morphometric measurement influencing δ^13^C and δ^15^N values (LME: P = 0.04, df = 1, F = 6.92, and P = 0.016, df = 1, F = 11.07, respectively), with increasing values for heavier individuals (increase at a rate of 1.23 ‰ kg^-1^ for both δ^13^C and δ^15^N). There were significant differences in blood δ^15^N values amongst the four clusters (LME: df = 3, F = 8.85, P<0.001) (Table 3). Post-hoc tests detected significant differences between clusters: individuals from cluster 1 had higher blood δ^15^N values than those from clusters 2 and 3, and lower values than those in cluster 4; in addition, individuals from cluster 4 had higher blood δ^15^N values than those in clusters 2 and 3. Feather δ^13^C and δ^15^N values averaged -15.2 ± 1.4 and 15.3 ± 1.2 ‰ (C:N mass ratio: 3.20 ± 0.05, n = 32). Large ranges in feather isotopic values were also observed, with differences between the lowest and highest values amounting to 6.7 and 3.9 ‰ in δ^13^C and δ^15^N, respectively. Importantly, both δ^13^C and δ^15^N values in blood and feathers were highly significantly positively correlated (92 ± 1% for δ^13^C, df = 1, F = 79.25 and 84 ± 1% for δ^15^N, df = 1, F = 37.24, respectively; Fig 5).

A total of 26 dietary samples were opportunistically collected, with a mean fresh mass amounting to 85 ± 43 g. Identified prey were benthic organisms including nine fish and one octopus species, the remaining identifiable prey items being errant polychaetes (Table 4). Overall the diet was dominated by *Notothenia cyanobrancha*, with *Lepidonotothen mizops* and *Harpagifer kerguelensis/spinosus* ranking second and third, respectively. Prey diversity was low with 85% of samples containing 1–3 prey species (maximum 7), but the dominant prey in samples differed between individuals. Repeat samples were obtained from 7 individuals; in 3 of them, the dominant prey remained consistent between the incubation and chick-rearing periods.

**Table 4:** Prey items found in regurgitate samples from Kerguelen shags.

Prey species	Prey group	Number of prey items	Proportion of prey items (%)	Number of individuals associated with each prey item	Proportion of individuals associated with each prey item (%)
*Muraenolepis marmoratus*	Fish	2	0.9	2	7.7
*Zanclorhynchus spinosus*	Fish	1	0.5	1	3.8
*Gobionotothen acuta*	Fish	3	1.4	1	3.8
*Lepidonotothen mizops*	Fish	32	14.4	6	23.1
*Notothenia cyanobrancha*	Fish	92	41.4	21	80.8
*Notothenia rossii*	Fish	1	0.5	1	3.8
*Paranotothenia magellanica*	Fish	14	6.3	10	38.5
Nototheniidae sp.	Fish	3	1.4	3	11.5
*Harpagifer kerguelensis/spinosus*	Fish	54	24.3	11	42.3
*Channichthys rhinoceratus*	Fish	1	0.5	1	3.8
Undetermined fish	Fish	1	0.5	1	3.8
Polynoidae sp.	annelid	13	5.9	9	34.6
*Benthoctopus thielei*	cephalopod	5	2.3	4	15.4

## Discussion

Using complementary methods corresponding to different timescales (bio-logging, stable isotopes [35]), the present study showed strong individual specialisations in a resident, benthic predator over different timescales (consecutive trips within a breeding stage, between stages of a single breeding season, and between the breeding and interbreeding periods). Importantly, four clusters were identified, corresponding to different strategies in terms of mean space use metrics and consistency, and related to differences in body masses and trophic levels.

Sexual dimorphism in birds is known to relate to differential niche utilization [54]. Kerguelen shags are sexually dimorphic and are temporally segregated with females foraging earlier in the day than males, and with males diving significantly deeper than females at some colonies [23]; these patterns appear to be widespread in the blue-eyed shag complex [29, 33, 55–59]. No differences in means for maximum dive depth, distances travelled and headings were found between sexes in the present study. Thus, differential niche utilization might be driven more by individual specialisations than sex in Kerguelen shags, although sex still influenced some aspects of behavioural consistency. Male and female shags in our study also lacked differences in both their δ^13^C and δ^15^N values, suggesting that males and females had similar habitats and diets, respectively.

Male and female Kerguelen shags in the present study did not differ in mean maximum dive depths, which contradicts other studies performed on species of the blue-eyed shag complex [20, 29, 30, 56, 57, 60]. The small sample size for birds equipped with dive recorders and the large inter- and intra-individual variation in depths in males might have been responsible for the lack of statistical significance in regards to the influence of sex on dive depths. The difference in consistency in maximum depths between males and females, however, is in line with values reported in [20, 56] who proposed differential degrees of individual specialisation within sexes as a mechanism for vertical niche partitioning. Harris et al. did not report differences in consistency between sexes in the depth of area-restricted search (ARS) areas but identified differences in consistency in other aspects of the foraging behaviour of the Imperial shags they instrumented; indeed females were more consistent in the maximum distances reached from the colony and the shore [33]. They suggested that these differences might be linked with sexual dimorphism, which constrains one sex more than the other and reduces their behavioural plasticity.

True individual specialisations (i.e. independent of sex, [6]) were emphasized in the present study. Individuals were indeed very repeatable in their headings to foraging zones, total distances travelled, and in dive depths, especially for females for which dive depth was the variable showing the highest repeatability. Fidelity of shags to a restricted diving depth range has been reported in other studies and was suggested to occur as a result of fidelity to specific food patches [7, 20, 56, 61], which is consistent with that observed in some individuals in the present study. Individuals sampled at incubation and a month later during the chick-rearing period were markedly consistent in their space use, exploiting the same foraging areas in both periods. The percentages of variance explained by the individual component reported in the present study are similar to those in [34] (e.g. 91% for maximum distances from the colony, 76% for depth of ARS), indicating those foraging metrics might show high repeatability within species of the blue-eyed shag complex.

The Kerguelen shags in the present study exhibited consistent feeding strategies, as indicated by stable isotope values, between stages within the breeding season but also between the breeding and inter-breeding periods. Other species from the blue-eyed shag complex show dietary specialisations that can be maintained over long timescales, consistent with the ability of some animals to adopt long-term behavioural strategies [3, 20, 32, 33].

A large number of seabird species have been shown to exhibit significant consistency in foraging strategies [9]. However, a significant degree of foraging consistency within a population does not necessarily mean that all individuals are consistent [17]. In our study, there were indeed differences in the behaviours exhibited by birds and in their consistency, which could not be explained by sex or breeding stage. Individuals tended to repetitively forage in the same areas and search for prey at the terminal part of their track in relatively restricted areas [18, 33]. This suggests they deliberately re-visited the same areas and, hence, that food patches exploited were localised and predictable in time and space [23, 33].

Similar to other species of the blue-eyed shag complex, Kerguelen shags are benthic divers and seafloor characteristics or bathymetric features could provide them with cues to memorize the location and quality of distinct foraging areas [7, 30, 62]. Alternatively, as shown in northern gannets (*Morus bassanus*), individuals might be responding differentially to environmental variables that are good proxies for prey type and abundance [5]. Returning to known profitable areas could reduce search time, and increase the efficiency of prey localization and capture as a result of experience [7]. Lastly, individual specialisations could be an important mechanism to reduce intra-specific competition for predators with a restricted foraging range, such as Kerguelen shags and other species of the blue-eyed shag complex, needing to feed their offspring at regular intervals [6, 10, 17, 18, 33, 63].

Rather than being explained by sex or stage, foraging strategies corresponding to different types of behaviours and levels of consistency were associated with prey of different trophic levels. Such dietary specialisations have been reported in Kerguelen and South Georgian shags [32] and have been matched to specialisation in foraging behaviour [33, 34, 62]. If prey types determine spatial use in these inshore divers, it is logical that they consistently prospect areas of similar characteristics (e.g. in terms of substrate or water depth), making them more consistent [33]. If preferred prey items have different ecological niches, individuals might differ in their consistency when exploiting specific prey.

Individual morphology did not appear to influence isotopic niche and there were no significant differences in morphometric measurements between the observed foraging strategies, confirming the existence of true specialisations (i.e. independent of discrete morphological groups, [6]). As there were no sex or breeding stage biases to foraging strategies, what drives individual prey preferences still remains unknown. Some studies have shown benefits of individual specialisations (e.g. better reproductive output, [4, 5, 64, 65]; body condition and survival, [65]), while others failed to do so in various contexts (e.g. survival and reproductive fitness, [62]; body condition, [17]). In the present study, there were some differences in body mass between individuals displaying the four identified foraging strategies, although these results should be interpreted with caution due to our small sample size. It was not possible to ascertain whether the different strategies were a consequence of body mass variation (e.g. potential physiological advantages in flying or diving [66]) or the differences in mass a consequence of the strategies employed (i.e. variation in strategy profitability); it seems, however, more likely for body mass to influence individual consistency than *vice versa* in birds that exhibit determinate growth. Heavier birds, such as birds from clusters 1 and 2, tended to fly farther, potentially indicating they were bringing back larger food loads, consistent with the optimal foraging theory. We suggest that the strategies identified arise as the best compromise for specific individuals given their intrinsic characteristics to respond to the variability of coastal resources [67].

In summary, the results of the present study have demonstrated strong individual specialisations in space use and diet across multiple temporal scales in a benthic forager, the Kerguelen shag, consistent with patterns reported for different species of the blue-eyed shag complex. Within the population, different foraging strategies could be highlighted and were associated with different levels of behavioural consistency and dietary choices. Sex, breeding stage and body measurements did not influence individual strategies. We show here the usefulness of using a combination of approaches–merging spatial, behavioural and dietary analyses–to investigate the links between foraging behaviour, its consistency and diet in individuals, which is essential to understand and accurately characterise ecological processes [35]. Future studies should investigate if such links are maintained in years of different environmental conditions and prey availabilities and whether age/experience influences consistency and individual specialisation. Furthermore, whether individual strategies confer specific advantages in terms of immediate foraging success and overall fitness should be determined.

## Supporting information

S1 Table Summary of dive metrics. (PDF)

S2 Table Summary of spatial use metrics. (PDF)

S3 Table Summary of morphometric measurements. (PDF)

S4 Table Summary of stable isotopes data. (DOCX)
