# Supplementary material for: Combined bio-logging and stable isotopes reveal individual specialisations in a benthic coastal seabird, the Kerguelen shag
Source: PLoS One. 2017 Mar 6;12(3):e0172278. doi: 10.1371/journal.pone.0172278 (PMC5338780; doi:10.1371/journal.pone.0172278)
Supplement: S1 Table — (PDF) [file pone.0172278.s001.pdf]

Diver number	sex	maximum depth (meters)		vertical distance travelled (meters)	
		Mean ± SD	CV	Mean ± SD	CV
1 (n=8 trips)	Male	55.1 ± 23.3	0.11	1053.0 ± 372.3	0.7
3 (n=9 trips)	Male	22.6 ± 3.7	0.04	1508.9 ± 503.0	0.33
8 (n=3 trips)	Male	69.8 ± 18.0	0.25	1697.4 ± 980.0	0.54
9 (n=9 trips)	Male	51.2 ± 17.9	0.2	949.1 ± 316.4	0.61
10 (n=7 trips)	Male	34.5 ± 12.7	0.15	1158.4 ± 437.8	0.47
12 (n=6 trips)	Male	2.3 ± 1.1	0.06	359.3 ± 146.7	0.72
2 (n=5 trips)	Female	27.6 ± 2.7	0.04	1371.4 ± 613.3	0.28
4 (n=4 trips)	Female	25.6 ± 3.9	0.15	1566.8 ± 783.4	0.44
5 (n=5 trips)	Female	19.9 ± 4.2	0.16	1183.7 ± 529.4	0.68
6 (n=5 trips)	Female	12.4 ± 2.9	0.07	1515.8 ± 677.9	0.48
7 (n=5 trips)	Female	52.6 ± 13.8	0.24	1326.6 ± 593.3	0.28
11 (n=10 trips)	Female	10.2 ± 2.8	0.25	1657.0 ± 524.0	0.56

dive duration (seconds)

Mean ± SD	CV
191.1 ± 68.8	0.14
100.9 ± 34.2	0.11
229.1 ± 126.9	0.16
179.6 ± 59.3	0.06
150.2 ± 58.0	0.14
22.0 ± 8.7	0.21
126.7 ± 56.5	0.04
128.3 ± 63.0	0.1
119.3 ± 55.6	0.14
86.1 ± 39.4	0.07
190.5 ± 85.7	0.12
59.6 ± 19.6	0.15
