# Supplementary material for: Combined bio-logging and stable isotopes reveal individual specialisations in a benthic coastal seabird, the Kerguelen shag
Source: PLoS One. 2017 Mar 6;12(3):e0172278. doi: 10.1371/journal.pone.0172278 (PMC5338780; doi:10.1371/journal.pone.0172278)
Supplement: S2 Table — (PDF) [file pone.0172278.s002.pdf]

Bird number	Trip number	sex	stage	Trip duration (hours)
1	1	female	incubation	5.67
1	2	female	incubation	6.95
1	3	female	incubation	6.39
2	1	female	incubation	3.40
2	2	female	incubation	0.75
2	3	female	incubation	6.36
2	4	female	incubation	4.50
3	1	male	incubation	1.05
3	2	male	incubation	1.38
3	3	male	incubation	1.27
3	4	male	incubation	1.29
3	5	male	incubation	3.26
3	6	male	incubation	1.71
3	7	male	incubation	5.55
3	8	male	incubation	1.13
3	9	male	incubation	0.79
3	10	male	incubation	3.69
3	11	male	incubation	2.86
3	12	male	incubation	1.70
5	1	male	incubation	2.63
5	2	male	incubation	7.95
5	3	male	incubation	6.57
5	4	male	incubation	8.61
5	5	male	incubation	8.07
6	1	male	incubation	2.83
6	2	male	incubation	9.70
6	3	male	incubation	0.68
6	4	male	incubation	7.30
6	5	male	incubation	8.57
6	6	male	incubation	7.60
6	7	male	incubation	5.17
7	1	female	incubation	5.84
7	2	female	incubation	6.11
7	3	female	incubation	6.35
7	4	female	incubation	6.70
8	1	female	incubation	4.40
8	2	female	incubation	8.17
8	3	female	incubation	6.04
8	4	female	incubation	6.51
8	5	female	incubation	7.19
10	1	female	incubation	7.46
10	2	female	incubation	7.86
10	3	female	incubation	7.90
10	4	female	incubation	7.49
10	5	female	incubation	5.16
10	6	female	incubation	2.27
11	1	female	incubation	5.81
11	2	female	incubation	6.79
11	3	female	incubation	6.11

11	4	female	incubation	6.66
11	5	female	incubation	7.83
12	1	male	incubation	8.16
12	2	male	incubation	6.68
12	3	male	incubation	4.19
12	4	male	incubation	9.17
12	5	male	incubation	6.59
14	1	male	incubation	5.98
14	2	male	incubation	8.20
14	3	male	incubation	9.53
14	4	male	incubation	8.65
15	1	male	incubation	3.87
15	2	male	incubation	3.80
15	3	male	incubation	7.78
15	4	male	incubation	6.01
15	5	male	incubation	8.60
15	6	male	incubation	9.21
16	1	male	incubation	4.82
16	2	male	incubation	9.36
16	3	male	incubation	8.47
16	4	male	incubation	3.46
16	5	male	incubation	3.28
16	6	male	incubation	5.86
16	7	male	incubation	5.26
16	8	male	incubation	6.12
16	9	male	incubation	7.10
18	1	female	incubation	5.86
18	2	female	incubation	5.26
18	3	female	incubation	6.12
18	4	female	incubation	7.10
20	1	female	incubation	6.47
20	2	female	incubation	7.34
20	3	female	incubation	0.70
20	4	female	incubation	6.53
21	1	male	chick-rearing	4.97
21	2	male	chick-rearing	8.26
21	3	male	chick-rearing	0.68
21	4	male	chick-rearing	5.13
21	5	male	chick-rearing	0.70
21	6	male	chick-rearing	5.74
21	7	male	chick-rearing	2.70
21	8	male	chick-rearing	8.20
12	6	male	chick-rearing	7.62
12	7	male	chick-rearing	2.94
12	8	male	chick-rearing	6.73
12	9	male	chick-rearing	4.00
12	10	male	chick-rearing	7.37
12	11	male	chick-rearing	8.83
12	12	male	chick-rearing	1.39
12	13	male	chick-rearing	9.11

12	14	male	chick-rearing	6.11
12	15	male	chick-rearing	4.59
12	16	male	chick-rearing	8.74
12	17	male	chick-rearing	3.35
23	1	female	chick-rearing	7.71
23	2	female	chick-rearing	6.18
23	3	female	chick-rearing	6.98
23	4	female	chick-rearing	5.43
23	5	female	chick-rearing	3.28
8	6	female	chick-rearing	5.65
8	8	female	chick-rearing	1.76
8	9	female	chick-rearing	4.82
8	10	female	chick-rearing	4.28
8	11	female	chick-rearing	5.35
8	12	female	chick-rearing	9.57
4	1	female	chick-rearing	6.78
4	2	female	chick-rearing	6.83
4	3	female	chick-rearing	2.75
4	4	female	chick-rearing	7.25
4	5	female	chick-rearing	2.38
4	6	female	chick-rearing	8.17
4	7	female	chick-rearing	6.47
4	8	female	chick-rearing	3.64
15	7	male	chick-rearing	10.00
15	8	male	chick-rearing	5.73
15	9	male	chick-rearing	1.51
15	10	male	chick-rearing	6.34
15	11	male	chick-rearing	0.74
15	12	male	chick-rearing	6.39
15	13	male	chick-rearing	1.83
1	4	female	chick-rearing	8.86
1	5	female	chick-rearing	7.75
1	6	female	chick-rearing	8.27
1	7	female	chick-rearing	8.30
1	8	female	chick-rearing	6.79
28	1	male	chick-rearing	6.56
28	2	male	chick-rearing	3.39
28	3	male	chick-rearing	5.18
28	4	male	chick-rearing	6.86
28	5	male	chick-rearing	3.59
28	6	male	chick-rearing	4.69
28	7	male	chick-rearing	5.17
28	8	male	chick-rearing	3.56
28	9	male	chick-rearing	4.29
16	10	male	chick-rearing	6.21
16	11	male	chick-rearing	6.21
16	12	male	chick-rearing	5.26
16	13	male	chick-rearing	7.49
16	14	male	chick-rearing	8.09
14	5	male	chick-rearing	9.31

14	6 male	chick-rearing	3.35
14	7 male	chick-rearing	3.80
14	8 male	chick-rearing	4.19
14	9 male	chick-rearing	1.08
14	10 male	chick-rearing	2.74
14	11 male	chick-rearing	4.69
14	12 male	chick-rearing	3.34
14	13 male	chick-rearing	6.36
14	14 male	chick-rearing	2.95
14	15 male	chick-rearing	6.04
14	16 male	chick-rearing	3.50
14	17 male	chick-rearing	4.33
14	18 male	chick-rearing	8.80
14	19 male	chick-rearing	7.37
14	20 male	chick-rearing	8.12
14	21 male	chick-rearing	4.89
14	22 male	chick-rearing	4.39
31	1 female	chick-rearing	6.21
31	2 female	chick-rearing	8.74
31	3 female	chick-rearing	2.32
31	4 female	chick-rearing	7.05
32	1 female	chick-rearing	8.22
32	2 female	chick-rearing	1.14
32	3 female	chick-rearing	9.75
32	4 female	chick-rearing	2.49
32	5 female	chick-rearing	4.68
34	1 female	chick-rearing	9.23
34	2 female	chick-rearing	7.33
34	3 female	chick-rearing	8.41
34	4 female	chick-rearing	8.52
34	5 female	chick-rearing	1.01
35	1 female	chick-rearing	5.88
35	2 female	chick-rearing	4.43
35	3 female	chick-rearing	3.86
35	4 female	chick-rearing	6.20
35	5 female	chick-rearing	7.04
36	1 male	chick-rearing	7.28
36	2 male	chick-rearing	2.16
36	3 male	chick-rearing	7.21
18	5 female	chick-rearing	6.49
18	6 female	chick-rearing	1.58
18	7 female	chick-rearing	4.20
18	8 female	chick-rearing	5.13
18	9 female	chick-rearing	13.98
18	10 female	chick-rearing	3.35
18	11 female	chick-rearing	18.12
18	12 female	chick-rearing	6.88
9	1 male	chick-rearing	2.69
9	2 male	chick-rearing	4.89
9	3 male	chick-rearing	5.63

9	4	male	chick-rearing	1.35
9	5	male	chick-rearing	6.53
9	6	male	chick-rearing	1.67
39	1	male	chick-rearing	10.72
39	2	male	chick-rearing	7.53
39	3	male	chick-rearing	1.94
39	4	male	chick-rearing	9.02
39	5	male	chick-rearing	3.84
39	6	male	chick-rearing	2.83
39	7	male	chick-rearing	5.51
39	8	male	chick-rearing	3.32
39	9	male	chick-rearing	2.91
40	1	male	chick-rearing	3.37
40	2	male	chick-rearing	3.75
40	3	male	chick-rearing	8.58
40	4	male	chick-rearing	6.36
40	5	male	chick-rearing	1.77
40	6	male	chick-rearing	5.24
40	7	male	chick-rearing	3.81
41	1	female	chick-rearing	6.33
41	2	female	chick-rearing	4.34
41	3	female	chick-rearing	11.18
41	4	female	chick-rearing	7.56
41	5	female	chick-rearing	3.05
41	6	female	chick-rearing	3.05
41	7	female	chick-rearing	4.55
41	8	female	chick-rearing	4.25
41	9	female	chick-rearing	1.47
41	10	female	chick-rearing	7.03
42	1	male	chick-rearing	2.33
42	2	male	chick-rearing	14.72
42	3	male	chick-rearing	2.90
42	4	male	chick-rearing	2.62
42	5	male	chick-rearing	3.88
42	6	male	chick-rearing	3.19

Total distance travelled (meters)	Maximum distances reached (kilometers)	bearing (°)
52.68	24.61	22.28
58.71	24.61	23.27
61.67	24.68	22.34
15.46	6.27	15.13
5.15	2.35	36.47
20.15	7.29	22.28
17.72	8.06	32.73
0.92	0.25	-19.75
0.99	0.48	24.25
2.33	1.05	93.44
3.01	1.36	178.83
4.34	1.30	94.01
2.91	0.83	64.91
3.23	1.22	100.23
2.07	0.65	76.13
2.50	0.78	65.09
1.74	0.70	68.82
4.42	0.79	80.50
2.63	0.52	55.31
12.69	5.42	98.88
34.84	13.68	41.42
19.14	6.53	126.03
18.34	5.54	104.55
29.52	9.20	58.60
31.30	14.15	45.07
45.92	18.02	35.68
1.89	0.79	115.34
54.04	22.57	33.02
51.18	22.20	32.80
58.63	23.50	32.26
26.14	10.54	62.84
11.50	4.08	30.78
14.91	5.57	24.53
18.94	7.20	19.51
19.09	7.18	23.52
23.93	7.99	47.98
35.00	7.81	39.52
37.57	14.80	30.47
25.65	8.89	37.70
31.79	12.01	38.65
37.51	13.61	28.87
25.70	7.05	21.57
25.82	7.75	25.02
27.66	8.11	20.28
21.76	7.52	30.21
13.37	5.24	49.38
32.36	14.10	32.37
32.92	13.82	31.58
31.49	13.78	37.29

36.25	14.17	40.21
31.86	13.94	32.08
74.61	26.60	37.66
49.69	18.40	29.05
37.51	16.98	28.60
61.98	24.94	25.69
40.31	16.81	28.23
31.97	8.65	80.92
25.93	8.68	76.68
32.04	8.47	82.73
34.48	9.70	109.14
26.93	12.39	96.81
22.73	10.31	86.98
50.24	21.85	73.08
29.77	13.25	86.45
40.48	15.67	81.94
45.59	17.48	84.93
18.63	6.83	113.32
48.22	20.21	34.91
27.74	10.90	66.56
19.77	8.48	73.99
16.70	7.12	111.28
16.81	3.10	164.26
8.14	1.99	151.64
10.02	2.08	163.88
12.81	2.40	159.41
16.81	3.10	164.26
8.14	1.99	151.64
10.02	2.08	163.88
12.81	2.40	159.41
39.10	18.09	26.54
34.06	15.75	26.38
3.43	1.74	97.92
39.27	18.35	26.80
71.07	14.34	51.52
96.14	20.75	47.20
32.78	7.89	77.94
111.11	22.27	39.61
35.46	8.58	72.26
92.91	20.92	43.96
66.48	16.24	52.65
100.68	20.96	44.22
30.29	13.53	24.44
6.75	2.88	48.67
31.78	14.94	27.50
29.71	13.87	27.76
34.72	15.60	28.19
37.34	16.91	29.31
6.92	3.32	53.64
37.19	16.82	28.51

36.92	17.48	28.25
33.49	15.59	28.24
53.39	24.31	24.57
32.22	15.35	27.81
48.70	19.75	32.63
25.40	10.71	43.47
35.16	15.77	36.76
32.92	14.24	40.65
23.29	10.51	42.73
17.94	7.87	53.38
8.97	7.35	0.00
18.50	7.35	53.90
18.20	7.48	47.76
20.63	7.30	50.96
39.46	15.83	35.34
23.01	8.91	142.90
28.33	8.86	143.31
21.34	8.74	143.17
25.46	8.66	146.33
20.53	8.72	145.26
29.30	9.03	144.37
23.36	8.26	143.78
19.23	8.78	140.97
42.86	19.43	78.75
47.38	15.29	105.15
20.85	9.14	118.74
45.29	19.48	100.41
14.10	6.80	106.66
58.05	25.27	75.30
25.95	11.93	91.90
44.79	18.73	26.37
40.81	18.56	24.85
44.62	18.75	26.57
49.39	21.02	24.36
40.51	18.17	24.91
29.25	12.47	39.45
26.71	12.57	35.62
27.92	12.44	39.16
29.31	13.11	34.38
29.47	12.34	41.13
26.58	12.48	39.65
30.30	12.56	42.87
26.69	12.46	39.56
28.57	12.43	40.18
24.98	10.13	57.69
23.41	10.60	62.76
18.97	7.80	98.65
31.91	14.11	47.59
33.39	14.33	48.60
30.30	10.20	75.96

25.34	9.60	71.53
19.21	8.23	95.31
20.36	8.59	93.77
13.89	6.68	94.20
16.65	7.54	113.71
25.26	9.00	86.38
17.50	7.96	108.87
21.48	8.63	110.82
15.96	7.34	114.81
24.04	8.83	76.85
17.14	8.20	86.55
25.27	9.74	73.79
11.46	4.83	-168.40
21.11	8.66	79.34
12.39	5.94	167.89
17.72	7.64	105.04
17.94	8.10	98.13
19.58	6.33	143.38
23.38	7.66	139.93
5.12	1.76	-152.91
13.71	3.72	156.16
52.49	21.47	27.57
4.86	1.65	127.96
50.72	21.07	29.06
9.45	3.83	94.53
14.44	4.10	129.78
38.55	13.81	26.95
39.44	15.19	26.46
37.18	14.03	27.44
43.59	15.25	28.17
5.48	2.17	-4.28
31.91	14.33	55.59
39.25	18.59	37.69
38.12	17.90	56.67
33.94	15.08	44.80
49.67	23.39	48.83
37.80	12.21	82.33
15.05	7.08	117.38
42.65	17.79	56.60
11.97	3.10	164.93
4.56	2.09	165.11
6.83	2.00	163.32
5.95	2.11	163.13
12.05	3.19	166.70
4.85	1.99	152.38
12.70	3.13	166.18
17.28	6.58	150.25
17.18	5.76	34.00
18.00	6.60	39.14
28.83	11.39	32.00

12.28	5.42	32.20
19.91	7.62	36.20
11.40	5.18	38.81
40.72	11.98	68.44
23.50	8.28	87.35
15.73	7.32	110.32
27.88	9.63	77.74
20.61	9.04	72.21
16.32	7.89	87.84
18.66	8.23	78.61
21.44	9.56	70.70
16.91	8.12	86.28
23.35	10.08	52.05
29.04	11.12	50.46
57.22	24.74	31.92
57.32	24.96	35.07
14.46	6.50	106.79
23.90	9.72	60.75
22.21	9.72	65.09
4.66	1.22	-164.86
6.05	2.02	14.84
12.71	2.38	29.28
8.89	2.08	20.75
2.81	1.10	-132.87
3.17	1.18	-168.21
7.15	2.37	28.75
4.34	1.24	16.89
2.95	1.13	22.45
5.74	1.20	25.78
4.49	1.42	-7.51
30.27	6.76	2.33
4.75	1.51	-14.83
4.74	1.34	-6.94
8.14	2.72	-36.41
4.30	1.40	-7.21
