# Supplementary material for: Combined bio-logging and stable isotopes reveal individual specialisations in a benthic coastal seabird, the Kerguelen shag
Source: PLoS One. 2017 Mar 6;12(3):e0172278. doi: 10.1371/journal.pone.0172278 (PMC5338780; doi:10.1371/journal.pone.0172278)
Supplement: S3 Table — (PDF) [file pone.0172278.s003.pdf]

| Bird numb | sex | Mean mass (kg) | Beak (millimeters) | Culmen (millimeters) |
|---|---|---|---|---|
| 1 | female | 2.5 | 15 | 47.3 |
| 2 | female | 2.1 | 13.4 | 49.1 |
| 6 | female | 2.3 | 14.2 | 52.6 |
| 7 | female | 2.2 | 13.4 | 52.3 |
| 8 | female | 2.2 | 16.9 | 52 |
| 9 | female | 2.3 | 12.3 | 49.7 |
| 14 | female | 2.3 | 13.4 | 48.8 |
| 15 | female | 2.2 | 13.7 | 50.7 |
| 17 | female | 2.3 | 12.5 | 51.7 |
| 18 | female | 2 | 11.9 | 52.2 |
| 20 | female | 2 | 12.7 | 49.5 |
| 21 | female | 1.9 | 12 | 50.5 |
| 22 | female | 1.9 | 12 | 48.6 |
| 23 | female | 1.9 | 12.1 | 50.9 |
| 28 | female | 1.9 | 12.4 | 47.9 |
| 3 | male | 2.6 | 15.7 | 53.4 |
| 4 | male | 2.5 | 15 | 53.1 |
| 5 | male | 2.4 | 13.1 | 53.3 |
| 10 | male | 2.6 | 14.3 | 55.6 |
| 11 | male | 2.4 | 13.5 | 56.6 |
| 12 | male | 2.6 | 12.7 | 40.9 |
| 13 | male | 2.5 | 15 | 51.1 |
| 16 | male | 2.2 | 14.8 | 55 |
| 19 | male | 2.4 | 14.2 | 54.4 |
| 24 | male | 2.5 | 12.7 | 53.9 |
| 25 | male | 2.4 | 13.6 | 54.2 |
| 26 | male | 2.6 | 13.5 | 52.1 |
| 27 | male | 2.3 | 15 | 50.7 |
| 29 | male | 2 | 13 | 49.7 |

| Wing (millimeters) | Tarsus (millimeters) | Head (millimeters) |
|---|---|---|
| 279 | 63.6 | 149 |
| 277 | 62.8 | 141 |
| 276 | 65.4 | 128.1 |
| 271 | 63.6 | 138.8 |
| 280 | 65.5 | 131.9 |
| 275 | 62.2 | 126.3 |
| 270 | 65 | 136.2 |
| 276 | 62.6 | 134.2 |
| 270 | 64.9 | 131 |
| 282 | 64.5 | 129.1 |
| 271 | 60.9 | 127.5 |
| 269 | 65 | 125 |
| 252 | 61.5 | 125 |
| 265 | 61.6 | 134 |
| 267 | 64.6 | 124.5 |
| 286 | 67.2 | 134 |
| 285 | 66.5 | 131.6 |
| 283 | 64.8 | 132.2 |
| 291 | 66.1 | 136.2 |
| 284 | 65.9 | 129.3 |
| 285 | 69.2 | 130.5 |
| 285 | 66.8 | 140.2 |
| 289 | 65.6 | 137 |
| 290 | 67.9 | 134 |
| 285 | 61.4 | 134 |
| 290 | 64.9 | 134 |
| 293 | 68 | 142 |
| 183 | 66.2 | 129.3 |
| 276 | 64.7 | 125.5 |
