# Supplementary material for: Combined bio-logging and stable isotopes reveal individual specialisations in a benthic coastal seabird, the Kerguelen shag
Source: PLoS One. 2017 Mar 6;12(3):e0172278. doi: 10.1371/journal.pone.0172278 (PMC5338780; doi:10.1371/journal.pone.0172278)
Supplement: S4 Table — (DOCX) [file pone.0172278.s004.docx]

| Bird | sex | stage | blood δ^13^C (‰) | blood δ^15^N (‰) | feather δ^13^C (‰) | feather δ^15^N (‰) |
| --- | --- | --- | --- | --- | --- | --- |
| 1 | female | incubation | -18.94 | 15.79 | -18.13 | 17.35 |
| 1 | female | chick-rearing | -18.17 | 15.27 |  |  |
| 2 | female | incubation | -14.68 | 16.17 | -13.88 | 16.53 |
| 3 | male | incubation | -19.97 | 16.49 | -20.06 | 17.39 |
| 4 | female | incubation | -18.25 | 13.54 | -16.78 | 13.74 |
| 4 | female | chick-rearing | -18.24 | 12.88 |  |  |
| 5 | male | incubation | -16.46 | 14.77 | -14.93 | 15.08 |
| 6 | male | incubation | -16.14 | 14.30 | -14.88 | 15.27 |
| 7 | female | incubation | -14.80 | 15.83 | -13.57 | 16.26 |
| 8 | female | incubation | -16.13 | 14.88 |  |  |
| 9 | male | incubation | -14.97 | 15.99 | -13.77 | 16.60 |
| 9 | male | chick-rearing | -14.44 | 15.27 |  |  |
| 10 | female | incubation | -15.41 | 15.46 | -14.71 | 16.35 |
| 11 | female | incubation | -15.70 | 14.89 | -14.70 | 14.42 |
| 12 | male | incubation | -17.89 | 15.79 |  |  |
| 12 | male | chick-rearing | -16.66 | 15.19 | -15.54 | 16.67 |
| 13 | male | incubation | -15.84 | 13.82 | -14.28 | 15.11 |
| 13 | male | chick-rearing | -15.75 | 13.41 |  |  |
| 14 | male | incubation | -15.90 | 13.48 | -14.45 | 14.56 |
| 14 | male | chick-rearing | -16.03 | 13.23 |  |  |
| 15 | female | incubation | -15.62 | 14.48 | -14.60 | 14.52 |
| 15 | male | chick-rearing | -15.44 | 13.93 |  |  |
| 16 | female | incubation | -14.95 | 15.52 | -13.32 | 16.48 |
| 16 | female | chick-rearing | -14.70 | 15.07 |  |  |
| 17 | female | incubation | -16.74 | 14.87 | -15.59 | 15.09 |
| 17 | female | chick-rearing | -16.70 | 13.99 |  |  |
| 18 | male | incubation | -15.83 | 13.32 | -14.65 | 14.21 |
| 19 | female | incubation | -18.03 | 15.28 | -16.68 | 16.01 |
| 20 | male | chick-rearing | -15.72 | 13.41 | -13.99 | 13.96 |
| 21 | female | chick-rearing | -16.33 | 14.50 | -14.93 | 15.46 |
| 22 | female | chick-rearing | -16.02 | 14.31 | -15.43 | 15.43 |
| 23 | male | chick-rearing | -15.70 | 14.20 | -15.33 | 14.98 |
| 24 | female | chick-rearing | -16.82 | 13.74 | -16.71 | 13.47 |
| 25 | female | chick-rearing | -16.16 | 14.20 | -16.51 | 14.46 |
| 26 | female | chick-rearing | -16.55 | 15.79 | -14.85 | 17.41 |
| 27 | female | chick-rearing | -15.99 | 13.29 | -14.29 | 14.00 |
| 28 | male | chick-rearing | -15.79 | 12.97 | -14.38 | 13.91 |
| 29 | male | chick-rearing | -15.52 | 13.55 | -13.64 | 14.82 |
| 30 | male | chick-rearing | -15.65 | 14.46 | -14.60 | 14.65 |
| 31 | female | chick-rearing | -15.31 | 15.54 | -15.73 | 16.66 |
| 32 | male | chick-rearing | -19.69 | 16.68 | -16.30 | 14.01 |
